# Reviewers

**DOI:** 10.1093/jncics/pkad057

**Published:** 2023-09-25

In celebration of Peer Review Week (September 25–29, 2023) we wish to thank the following reviewers for their generous dedication of time in evaluating manuscripts submitted to *JNCI Cancer Spectrum* during the past year. Those who were our top reviewers, turning in 3 or more reviews on time, are indicated with asterisks.

Balazs Acs

Prajakta Adsul

Amir Alishahi Tabriz

Andrea Arfe

Elizabeth Arthur

Dagfinn Aune

Tarah Ballinger

Shrujal Baxi

Peter Beitsch

Tanios Bekaii-Saab

Al Benson

Marianne Berwick

Ana Best*

Sheena Bhalla

Jiang Bian

Prunella Blinman

Natalie Bradford

Tara Brinkman

Justin Brown

Harry Burke

Elizabeth Cahoon

Rikki Cannioto*

Ying Cao*

Cheng Cheng

Ting-Yuan David Cheng

Li Cheung

Jeffrey Clark

Graham Colditz

Norah Crossnohere

Jennifer Croswell

Todd DeWees

Adam Dicker

Christina Dieli-Conwright

Michaela Dinan*

Kevin Dobbin*

Barbara Dunn

Christen Elledge

Edward Ellerbeck

Cathy Eng

Katherine Enright

Anessa Foxwell

Lisa Gallicchio

Apar Kishor Ganti

Patricia Ganz

Franklin Gaylis

Ann Geiger

Alessandra Giovagnoli

Bonnie Gould Rothberg

Amanda Graham

Peter Grimison

Shalina Gupta-Burt

Michael Halpern

Summer Han

Sheetal Hardikar

Elizabeth Hatch

Julie Heimbach

Kathy Helzlsouer

Jordan Holmes

William Hsu

Xin Hu

I-Chan Huang

Matthew Hudson*

Lynn Ibekwe

Stephanie Jarosek

Katie Jones

Himanshu Joshi

Corinne Joshu

John Kairalla

Elizabeth Kantor

Aaron Katz*

Kelley Kidwell

Belinda Kiely

Gretchen Kimmick*

Andrea Knezevic

Bogda Koczwara

Panagiotis Konstantinopoulos

Tzy-Mey Kuo*

Carlo La Vecchia

Chris Labaki

Nicola Lawrence

Michael Leapman

Kyuwan Lee

Wendy London

Holli Loomans-Kropp

Philip Lupo

Hung Luu

James Lynam

Clement Ma

Margaret Madeleine

Sally Maliski

Deborah Mayer

Gina Mazza

Tomer Meirson

Tamara Miller

Aaron Mitchell*

Kathi Mooney

Motomi Mori*

Kent Mouw

Megan Mullins

Steven Narod

Michelle Naughton

Sarah Nechuta

Heather Neuman

Hazel Nichols

Leticia Nogueira

Christopher O'Callaghan

Nitin Ohri

Haitao Pan

I-Wen Pan

Electra Paskett

Liem Phan

Shelley Potter

Sheila Prindiville

Anne Reiner*

Philip Rosenberg

B. R. Rosser

Lawrence Rubinstein

Subhrajit Saha

Ilyas Sahin

Elizabeth Salerno

John Salsman

Thomas Samaille

A. Oliver Sartor

Carolyn Schwartz

Priyanka Sharma

Sanjay Shete

Jianxin Shi

David Shibata

Katrin Sjoquist

Andrzej Slominski

Kalyani Sonawane

Susan Steck

Shane Stecklein

Thomas Stinchcombe

Shivani Sud

Weijing Sun

Zhuoxin Sun

Ray Tan

Sarah Temkin

Kim Templeton

Melissa Troester

Justin Trogdon

Joseph Unger

Janette Vardy

Deepthi Varma

Anuradha Vasista

Samuel Volchenboum

Eleanor Walker

Matthew Wallis

Kai Wang*

Jenny Wei

Kevin Weinfurt

Jennifer Weiss

Karen Wernli

Nicholas Wilcken

James Willey

Jan Wohlfahrt

Juan Yang

Robert Yarchoan

Dougie Zubizarreta

